# Effects of Multicomponent Exercise on Pain and Biopsychosocial Outcomes in Adults with Cancer: A Systematic Review

**DOI:** 10.3390/healthcare13222842

**Published:** 2025-11-09

**Authors:** Leidy Tatiana Ordoñez-Mora, Juan Fernando Gómez-Gómez, Mateo Marquez-Bustamante, Ilem D. Rosero, Giovanna Patricia Rivas-Tafurt, Jose Luis Estela-Zape

**Affiliations:** 1 Physiotherapy Program, Faculty of Health, Universidad Santiago de Cali, Santiago de Cali 760035, Colombia; mateomarquezbustamante@gmail.com (M.M.-B.); ilem.rosero00@usc.edu.co.com (I.D.R.); jose.estela00@usc.edu.co (J.L.E.-Z.); 2 Internal Medicine Specialization Program, Department of Health, Universidad Santiago de Cali, Santiago de Cali 760035, Colombia; juan.gomez51@usc.edu.co (J.F.G.-G.); giovanna.rivas@clinicadeoccidente.com (G.P.R.-T.); 3 Research and Education Group GIEDCO, Clínica de Occidente S.A, Santiago de Cali 760046, Colombia

**Keywords:** exercise, neoplasms, pain measurement, quality of life

## Abstract

Background/Objective: Cancer significantly impairs physical function and quality of life. Exercise has gained recognition as a therapeutic strategy; however, its long-term efficacy in terms of multidimensional outcomes in patients with cancer remains underexplored. This review aimed to evaluate and summarize the available evidence regarding the effects of multicomponent exercise programs on pain and psychosocial outcomes in individuals with cancer. Methods: A systematic review was conducted in accordance with PRISMA guidelines and registered in PROSPERO (CRD42022321183). Comprehensive searches were performed in MEDLINE, OVID, LILACS, Scopus, PEDro, OTseeker, The Cochrane Library, EBSCO, and Google Scholar, without date restrictions. Search terms included “exercise” and “cancer.” Only randomized controlled trials (RCTs) were included. Methodological quality and risk of bias were assessed using the PEDro scale. Results: Sixteen randomized controlled trials met the inclusion criteria. Multicomponent exercise programs combining aerobic, resistance, and flexibility training significantly improved muscle strength, balance, and quality of life. Several studies reported meaningful reductions in pain intensity and interference, whereas others found no significant changes. Overall, exercise interventions demonstrated superior effects compared with usual care or educational controls across physical and psychosocial outcomes. Conclusions: Multicomponent exercise is a safe and feasible intervention for adults with cancer, including those with advanced disease or complex clinical profiles. Evidence supports consistent benefits in physical function and quality of life, with partially consistent yet favorable effects on pain. Programs integrating multiple exercise modalities appear most effective and should be considered as part of comprehensive oncological care to enhance therapeutic outcomes and long-term well-being.

## 1. Introduction

Cancer is the leading cause of death worldwide and the second most frequent cause of morbidity and mortality in the Americas, following cardiovascular diseases. In 2020, nearly 10 million deaths globally were attributed to cancer. In the Americas, approximately 4 million new cases were diagnosed, and 1.4 million deaths were reported due to this disease [1,2]. According to GLOBOCAN [3], the most common cancers in terms of new cases across both sexes and all age groups were breast (11.7%), lung (11.4%), colorectal (10%), and prostate cancer (7.3%). The leading causes of cancer-related mortality were lung (18%), colorectal (9.4%), liver (8.3%), and gastric cancer (7.7%) [1,3]. Approximately 57% of new cancer cases and 47% of cancer-related deaths occurred in individuals aged 69 years or younger [3].

The primary causes of cancer stem from interactions between individual genetic predispositions and three categories of external agents: physical, chemical, and biological carcinogens [3]. About one-third of cancer-related deaths are attributable to modifiable risk factors, including tobacco use, high body mass index, alcohol consumption, low intake of fruits and vegetables, and physical inactivity [1,2]. Cancer mortality can be reduced through early diagnosis and timely treatment, as early detection increases treatment success rates, improves survival, and decreases morbidity [2].

Cancer-related symptoms include fever, severe fatigue, weight loss or feeding difficulties, pain, swelling or palpable masses, and abnormal bleeding. Tumors may also secrete substances that alter energy metabolism or elicit immune responses, contributing to these manifestations. Many of these symptoms are nonspecific and may be associated with other conditions or with adjuvant therapies such as chemotherapy or radiotherapy [4]. Physical complications, including fatigue, pain, and functional impairments, substantially affect daily activities [5]. Pain is a major determinant of reduced quality of life, frequently associated with sleep disturbances, social withdrawal, anorexia, and decreased physical activity [6]. Alterations in body image and sexual function further contribute to emotional distress [7,8].

Clinically significant stress, depression, anxiety, and deterioration in quality of life are also common after cancer treatment [9,10]. Proposed mechanisms include psychosocial distress related to the disease, such as heightened fear of recurrence [11], concerns about family and financial burden [12], and difficulties reintegrating into social life [13]. Pain perception is modulated by complex interactions among biological variables—including gonadal hormones, genetic factors, differences in nociceptive processing pathways, and structural or functional variations in the central nervous system [14]—as well as psychosocial variables such as depression, anxiety, catastrophizing, cultural background, social learning, perceived relevance of pain, sex, age, and perceived social support [15].

Reducing pain and improving psychosocial well-being to optimize health-related quality of life (HRQoL) is a key objective across all phases of cancer management and survivorship [16]. Physical exercise has been proposed as an effective adjunct to cancer treatment, demonstrating multiple benefits for intrinsic tumor responses and cancer-related symptoms [17]. Additionally, exercise may reduce the risk of cancer recurrence [18,19]. Recent evidence suggests that multicomponent exercise programs defined as planned, structured, and repetitive interventions that combine more than one type of exercise, such as aerobic, resistance, balance, and flexibility training, are safe and well tolerated by patients with cancer when appropriately designed and supervised. The effectiveness of such interventions is strongly influenced by the planned intensity, frequency, and duration of the combined exercises. Beyond safety, physical exercise has demonstrated substantial benefits in this population, including reductions in cancer-related fatigue, improvements in cardiorespiratory fitness, muscle strength, and functional capacity, as well as enhancements in psychological well-being and overall quality of life. Exercise has also been associated with better treatment tolerance, fewer adverse effects, and potential improvements in survival outcomes. Nevertheless, current evidence remains limited, and further comprehensive studies are required to determine the most effective strategies for structuring multicomponent programs in this population [20,21,22]. Against this background, the aim of this systematic review was to evaluate and summarize the available evidence regarding the effects of multicomponent exercise programs on pain and psychosocial outcomes in individuals with cancer.

## 2. Materials and Methods

This study followed the Cochrane Collaboration guidelines for systematic reviews [23] and the PRISMA checklist criteria [24]. The protocol was registered in the International Prospective Register of Systematic Reviews (PROSPERO) under ID CRD42022321183 (https://www.crd.york.ac.uk/prospero/display_record.php?ID=CRD42022321183, accessed on 6 November 2025).

### 2.1. Eligibility Criteria

Studies were selected based on the PICO framework, which is detailed as follows:

P (Population): Adult patients diagnosed with cancer or cancer survivors.

I (Intervention): Multicomponent exercise refers to programs that incorporate more than one type of exercise, most commonly strength, balance, and aerobic or endurance training. Additional components may include flexibility, coordination, power, or joint mobility, depending on the intended therapeutic goals and the scope of the intervention. For this review, inclusion criteria required the implementation of at least three combined exercise modalities [25,26,27].

C (Comparison): Conventional management or other non-pharmacological interventions.

O (Outcomes): Pain, catastrophizing, depression, disability index, life expectancy, quality of life, physical function, and pharmacological behavior.

Study type: Randomized controlled trials (RCTs) were included.

Exclusion criteria: Studies with interventions involving fewer than three exercise components or those incorporating additional non-exercise modalities were excluded.

### 2.2. Information Sources and Search Strategy

Searches were conducted in MEDLINE, OVID, LILACS, Scopus, PEDro, OTseeker, The Cochrane Library (Cochrane Central Register of Controlled Trials), EBSCO, and Google Scholar. The primary search terms were “exercise” and “cancer.” The complete search strategy is detailed in Appendix A: “(((exercise[Title/Abstract]) OR (exercise therapy[MeSH Terms])) AND (multicomponent[Title/Abstract])) AND (cancer[Title/Abstract]).” No language or publication date restrictions were applied. Searches were concluded on 10 March 2025.

### 2.3. Study Selection

A calibration process was implemented for study selection. Two independent reviewers screened the records in a blinded manner. Titles and abstracts were assessed using an online reference management system. Duplicate records were identified and removed with EndNote. Rayyan was used to support blinded screening of titles and abstracts and to flag potentially ineligible studies based on predefined filters. All outputs from these tools were verified by the reviewers to ensure accuracy. Articles were included if both reviewers reached agreement; disagreements were resolved by a third blinded reviewer. Eligibility criteria were applied during full-text screening for final selection. Any discrepancies regarding eligibility, quality assessment, or data extraction were resolved by consensus.

### 2.4. Data Collection Process

Data extraction was conducted independently using a standardized Excel form. Extracted variables included first author and year, study design, country, sample size, age, cancer type and stage, time since diagnosis, study objective, use of pharmacological treatment, description of multicomponent exercise modalities, comparator or additional interventions, measurement scales, and outcomes related to pain, catastrophizing, depression, disability index, life expectancy, quality of life, physical function, adherence, and adverse events. Mean and standard deviation values were extracted for all reported outcomes. Data accuracy was confirmed by all authors.

### 2.5. Quality Assessment and Risk of Bias

The methodological quality of the included studies was evaluated using the PEDro scale [28]. Studies with a score ≥ 6 were eligible for inclusion. The scale assesses random allocation, allocation concealment, baseline comparability, blinding of participants, therapists, and assessors, completeness of outcome data for at least 85% of participants in one key outcome, intention-to-treat analysis, and between-group statistical comparisons with corresponding results.

## 3. Results

**Figure 1:**
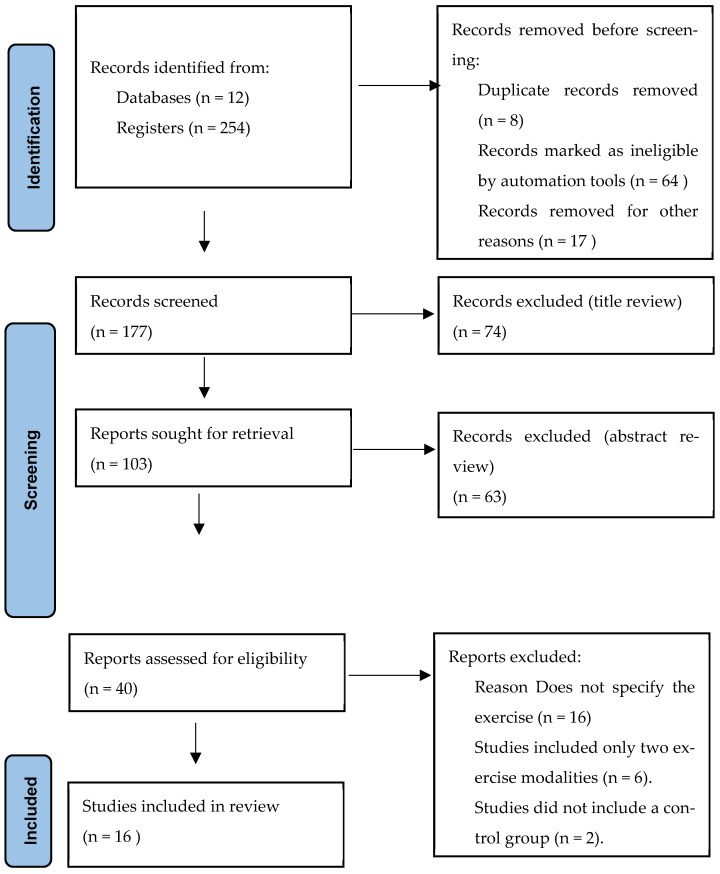
Flow diagram of study selection process. Source: Page MJ et al. BMJ 2021;372:n71. https://doi.org/10.1136/bmj.n71 [24].

The systematic search across 12 databases retrieved 254 records. After removing 8 duplicates and excluding 64 records marked as ineligible by automation tools, 177 records remained for title and abstract screening. Of these, 74 were excluded at the title review stage and 63 at the abstract review stage. Consequently, 40 articles were assessed in full text, of which 16 met the inclusion criteria and were included in the final analysis (Figure 1 and Table 1).

### 3.1. Description of the Intervention

A total of 10 studies compared the intervention with conventional care, while 6 used alternative exercise modalities as comparators. Conventional care generally consisted of pharmacological management combined with routine clinical recommendations. Alternative comparators included stretching or relaxation programs, standard hospital care, home-based exercise, and impact-based exercise.

The interventions were delivered as multicomponent exercise programs incorporating aerobic, resistance, balance, flexibility, and strength training. Aerobic training involved walking, cycling, or treadmill exercise; resistance training was performed with elastic bands, free weights, or machines; and balance and flexibility components included pelvic floor exercises, yoga, and stretching. Intervention periods ranged from 8 to 12 weeks, with sessions of 60 to 90 min conducted two to three times per week.

Aerobic training was included in 100% of interventions, most frequently as walking [29,30,31,32,33,34,35,36,37,38,39,40,41,42,43,44]. Aerobic intensity was monitored via heart rate monitors and ratings of perceived exertion (RPE). All studies incorporated strength training [29,30,31,32,33,34,35,36,37,38,39,40,41,42,43,44], employing bodyweight, elastic bands, or external weights. Flexibility training was also universal, typically performed through stretching at the end of sessions [29,30,31,32,33,34,35,36,37,38,39,40,41,42,43,44]. Additionally, two studies included pelvic floor training [31,36], one study reported respiratory training components [35], and four studies included balance training elements [33,40,41,42].

### 3.2. Description of Cancer Patient Characteristics

The most frequently reported cancer type was breast cancer [30,32,37,38,39,42,44], followed by prostate cancer [31,36,43], lung cancer [29,35], colorectal cancer [29,33], and mixed cancer populations [30,34,41]. Regarding disease stage, several studies included patients with advanced disease (stage IV) [29,33,43], while others enrolled patients across stages I–IV [38,39,40,41]. Populations comprised cancer survivors [30,31,32,34] and patients undergoing radiotherapy and/or chemotherapy [34]. Most studies included participants at least six months post-treatment.

### 3.3. Description of the Main Results of the Study

#### 3.3.1. Pain Outcomes

Studies evaluating multicomponent exercise interventions in patients with cancer have reported heterogeneous effects on pain outcomes. Cheville et al. (2013) found no significant changes in pain intensity measured by the Numeric Rating Scale (NRS), with similar reductions in both groups (IG: −0.62 ± 2.59 vs. CG: −0.50 ± 2.01; *p* = 0.87) [29]. Conversely, Reis et al. (2018) observed significant improvements in multiple pain domains using the Brief Pain Inventory (BPI), including total pain (*p* = 0.0047), general intensity (*p* = 0.0082), worst (*p* = 0.0284) and least pain (*p* = 0.0365), as well as pain interference in daily life (*p* = 0.0201) [30]. Similarly, Mardani et al. (2021) reported a significant decrease in pain assessed by the EORTC QLQ-C30 (IG: 40.47 ± 16.31 vs. CG: 28.24 ± 15.84; *p* = 0.002) [31], whereas Zopf et al. (2015) found no significant between-group differences in the same instrument (IG: −2.37 points; CG: +3.33 points; both *p* > 0.05) [36]. In another trial, Do et al. (2015) demonstrated a substantial reduction in EORTC QLQ-C30 pain scores following an exercise program (IG pre = 40.9 ± 28.1; post = 19.4 ± 13.6; *p* < 0.001), with no significant change in controls [37]. Among cancer survivors, Fernández-Rodríguez et al. (2023) reported a trend toward pain reduction on the VAS and EQ-5D pain domains after a home-based multimodal program, though these differences were not statistically significant (*p* = 0.334) [41]. In contrast, Haines et al. (2010) identified a significant improvement in EQ-5D VAS scores favoring the intervention group (mean difference = 10.08; 95% CI 2.84–17.32; *p* = 0.006) [42]. Finally, Galvão et al. (2018) reported no between-group differences in bone pain assessed with the FACT-BP (*p* = 0.507) [43]. Collectively, these findings indicate that while several trials demonstrate clinically meaningful improvements in pain perception and interference, the overall evidence remains mixed, likely due to differences in cancer type, intervention components, and assessment instruments (see Table 2).

#### 3.3.2. Disability Outcomes

Regarding disability, a study using the World Health Organization Disability Assessment Schedule 2.0 (WHODAS 2.0) in patients with active cancer found no significant differences between groups [35]. The intervention included home-based aerobic exercise (30 min, ≥3 days/week, moderate-to-high intensity based on Borg 13–16 and target heart rate), resistance training for major muscle groups (2 sessions/week, 3 sets of 10–12 repetitions), stretching with elastic bands, and respiratory training (10 min, twice daily). Despite this structured multicomponent approach, changes in disability were not significant (WHODAS 2.0 mean difference −1.0, 95% CI −2.4 to 0.4; *p* = 0.152) (see Table 2).

#### 3.3.3. Depression Outcomes

In cancer survivors, exercise interventions consistently demonstrated significant improvements in depressive symptoms compared with educational or usual care controls. In one supervised program [34], participants engaged in two 60-min sessions per week combining aerobic (20 min, 65–90% HRmax) and resistance training (progressing from 2 × 12 to 4 × 6 repetitions across upper and lower body exercises), complemented by encouragement to achieve 150 min/week of additional aerobic activity. Compared with the home-based group receiving only educational materials and telephone follow-up, the supervised group showed a marked reduction in HADS-D scores at week 12 (baseline 6.89 ± 4.20 to 4.00 ± 2.40), while the control remained unchanged (baseline 7.22 ± 2.49 to 7.67 ± 3.61). Similarly, another study [35] comparing exercise with educational interventions reported significant reductions in depressive symptoms favoring the exercise group. Furthermore, a multimodal supervised exercise program during radiotherapy and chemotherapy [44] produced greater improvements than routine nursing care, with significant between-group differences at mid- and post-intervention (HADS-D: t = −3.054, *p* = 0.003; t = −2.437, *p* = 0.019). Collectively, these findings highlight the robust effect of exercise, particularly supervised and multimodal programs, in reducing depression among cancer survivors.

#### 3.3.4. Quality of Life Outcomes

Quality of life outcomes were assessed in fourteen trials using validated instruments such as the EORTC QLQ-C30 [31,36,37,40,42,44], FACT-G and related subscales [29,33,38,41], SF-36 [34], and EQ-5D [41,42]. Overall, multicomponent exercise interventions produced consistent improvements in quality of life compared to usual care or educational controls, although the magnitude of change varied across studies. Cheville et al. (2013) observed significant gains in mobility (*p* = 0.002), but not in global FACT-G scores (*p* = 0.54) [29]. In contrast, Mardani et al. (2021) reported marked improvements in global health status measured by the EORTC QLQ-C30 (IG pre 60.19 ± 13.95, post 72.57 ± 11.63; CG pre 61.12 ± 14.10, post 63.40 ± 12.80; *p* < 0.001) [31]. Similarly, Wang et al. (2021) found significant gains in the physical well-being subscale of FACT-ES (*p* = 0.023) [32], and Zimmer et al. (2018) reported an increase in FACT-G total scores favoring the intervention group (*p* = 0.028) [33].

Improvements were also observed in mental health and emotional function domains. Levin et al. (2018) reported significant enhancements in the SF-36 Mental Health Composite after 12 weeks of exercise compared to the control group (*p* = 0.005) [34]. Do et al. (2015) demonstrated substantial improvements in global health status using the EORTC QLQ-C30 (IG pre 58.0 ± 18.6; post 87.3 ± 13.7; CG pre 59.5 ± 17.9; post 61.0 ± 17.5; *p* < 0.001) [37], while Shinde et al. (2024) observed increases in FACT-B scores (*p* = 0.001) [38]. Fernández-Rodríguez et al. (2023) also reported significant improvements in total FACT scores following a home-based multimodal program (*p* = 0.036) [41].

In patients with active cancer, Haines et al. (2010) documented significant gains in global health on the EORTC QLQ-C30 favoring the exercise group (*p* = 0.04) [42], while Zhang et al. (2023) found significant benefits in functional, emotional, and social functioning, as well as reductions in fatigue and insomnia (all *p* < 0.05) [44]. Conversely, some studies, such as Zopf et al. (2015) and Ferrara et al. (2025), reported non-significant changes in overall quality of life [36,40]. Collectively, these findings indicate that multicomponent exercise programs typically integrating aerobic, resistance, and flexibility training are associated with meaningful improvements in both general and cancer-specific quality of life, particularly in physical, emotional, and global health domains (see Table 2).

#### 3.3.5. Physical Function Outcomes

Across the included studies, multicomponent exercise interventions produced significant improvements in several indicators of physical function. Reis et al. (2018) reported higher aerobic capacity and handgrip strength in the intervention group compared with controls (VO_2_max: 20.68 ± 2.50 vs. 14.80 ± 2.46; *p* = 0.0001; handgrip: 24.79 ± 6.77 vs. 21.71 ± 7.44; *p* = 0.0001) [30]. Similarly, Wang et al. (2021) observed a significant increase in step counts during the 2-min step test (*p* = 0.036) [32], and Zimmer et al. (2018) demonstrated significant gains in leg press strength (*p* = 0.011) but no between-group differences in 6-min walk distance (*p* = 0.432) [33].

In patients with active cancer, Ferrara et al. (2025) reported improvements in lower limb strength (leg press 1RM: *p* = 0.153; knee extension 1RM: *p* = 0.018) and global performance measured by the SPPB (IG: 2.30 vs. CG: 0.38; *p* = 0.002) [40]. Fernández-Rodríguez et al. (2023) also found significant between-group differences favoring the intervention in total SPPB scores (6.21 ± 2.99 vs. 4.42 ± 3.03; *p* = 0.045) and gait performance (*p* = 0.035) [41]. Galvão et al. (2018) reported a significant increase in leg extension strength (mean difference 6.6 kg; 95% CI 0.6–12.7; *p* = 0.033) [43].

In breast cancer patients (stages I–III), Shinde et al. (2024) [39] observed significant improvements in lower limb function and aerobic performance following a multicomponent exercise intervention. The intervention group showed a notable increase in the sit-to-stand test (from 12.96 ± 4.24 to 17.12 ± 7.39; *p* = 0.0002) and in the 12-min walk test (from 1242.73 ± 205.68 m to 1309.37 ± 167.35 m; *p* = 0.008), while no significant changes were detected in the control group (*p* > 0.90). These findings further support the effectiveness of combined exercise modalities in enhancing functional capacity among individuals with cancer.

Other studies reported variable effects on aerobic capacity and general performance. Levin et al. (2018) found no between-group differences in 400-m walk performance (*p* = 0.466) [34], and Haines et al. (2010) observed non-significant differences in 6MWT distance (*p* = 0.34) and grip strength (*p* = 0.48) [42]. Conversely, significant within- and between-group improvements in physical function subscales were observed in studies using quality-of-life instruments, including EORTC QLQ-C30 (*p* < 0.001) [36,37]. Overall, exercise interventions combining aerobic, resistance, and flexibility training demonstrated consistent benefits in muscle strength, balance, and physical performance, while changes in aerobic capacity were heterogeneous across studies (see Table 2).

#### 3.3.6. Additional Findings

Most studies did not evaluate overall survival, focusing instead on functional outcomes. Some reported indirect indicators; for instance, Zimmer et al. (2018) [33] observed maintenance of neuropathic symptoms and muscle strength in patients with metastatic colorectal cancer receiving the intervention, whereas the control group demonstrated deterioration, indicating potential preservation of function in advanced disease.

Adherence was generally reported as acceptable to high, ranging from 70.8% to 100%. Several studies documented adherence around 80% (e.g., 76.9%, 80%, 80.5%, and 93.3%). In some cases, exact percentages were not specified, but compliance was described as good. Reported strategies to promote adherence included shorter interventions and regular follow-up, although consistency in reporting was limited.

No study reported outcomes related to catastrophizing. Life expectancy was primarily used for patient characterization, and no findings were presented regarding pharmacological treatment.

### 3.4. Risk of Bias Assessment

**Figure 2:**
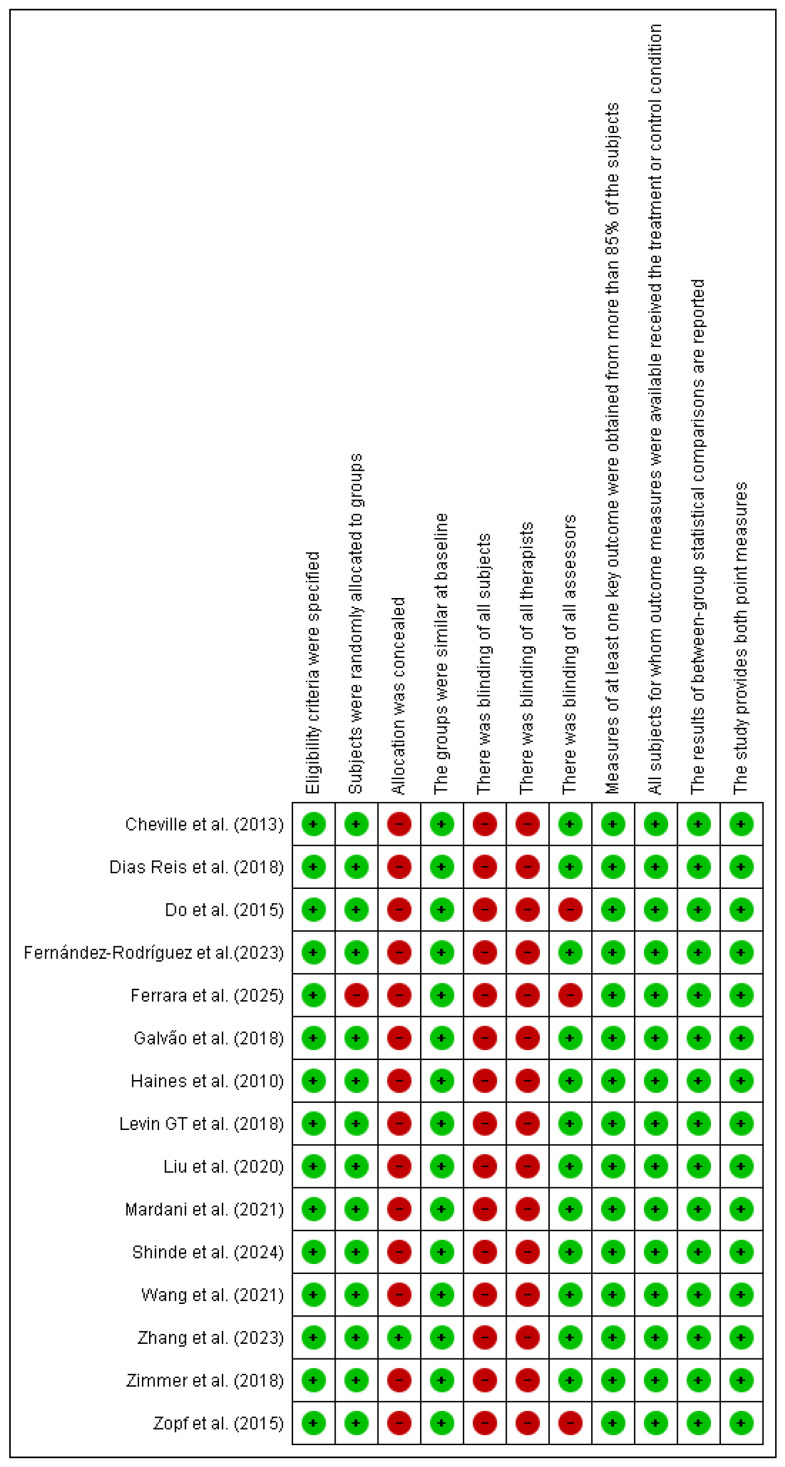
Risk of bias Pedro scale. Source: Risk of bias using the Pedro scale (developed with Revman 5.3) Abbreviations: Green: Low risk Red: High risk. Cheville et al. (2013) [29], Reis et al. (2018) [30], Mardani et al. (2021) [31], Wang et al. (2021) [32], Zimmer et al. (2018) [33], Levin GT et al. (2018) [34], Liu et al. (2020) [35], Zopf et al. (2015) [36], Do et al. (2015) [37], Shinde et al. (2024) [38,39], Ferrara et al. (2025) [40], Fernández-Rodríguez et al. (2023) [41], Haines et al. (2010) [42], Galvão et al. (2018) [43], Zhang et al. (2023) [44].

The risk of bias assessment, using the PEDro scale [28] identified the absence of blinding of participants and therapists as the main methodological limitation, which is inherent to exercise-based interventions. In addition, three studies did not implement blinding of outcome assessors, which may have introduced detection bias and affected the reliability of the findings. Nevertheless, other PEDro criteria, including randomization and intention-to-treat analysis, were consistently fulfilled. Overall, the methodological quality of the included studies is best characterized as moderate (see Figure 2).

## 4. Discussion

Consistent with global cancer prevalence patterns, the most frequently reported cancer types in the included studies were breast cancer (7 studies) and prostate cancer (3 studies), primarily in advanced stages and under active treatment.

Multicomponent exercise interventions were implemented across various cancer types and stages, with benefits observed in both early and advanced disease. Programs generally combined aerobic, resistance, flexibility, balance, and strength training, tailored to patient characteristics and treatment status. Aerobic exercise was consistently included, most often walking, cycling, or treadmill sessions, performed typically 2–3 times per week for 20–30 min per session, at moderate-to-vigorous intensity (65–90% HRmax or Borg 13–16), with some protocols encouraging the accumulation of 150 min/week of additional aerobic activity. Resistance training was conducted twice weekly, using elastic bands, free weights, or machines, with progressive overload (e.g., starting at 2 sets of 12 repetitions and advancing up to 4 sets of 6 repetitions) and targeting both upper and lower body muscle groups. Balance training was incorporated particularly in older adults and those with functional limitations, including static and dynamic exercises to improve stability, gait, and fall prevention. Flexibility exercises were usually included at the end of each session through stretching of major muscle groups. Additional disease-specific components included pelvic floor training in prostate cancer, aiming to improve continence and functional outcomes. Overall, interventions commonly lasted 8–12 weeks, with supervised 60–90-min sessions delivered two to three times per week, demonstrating the feasibility and effectiveness of structured multicomponent approaches in oncological populations.

For breast cancer, several studies reported consistent improvements in quality of life, fatigue, physical function, and psychological outcomes. Overall, multicomponent exercise interventions were associated with beneficial effects on pain, quality of life, and functional capacity in patients with active cancer and survivors. In particular, stage IV cancers including breast, and lung cancer demonstrated benefits from multicomponent exercise in reducing pain, lowering disability, and improving functional status and quality of life.

These findings align with Zhang et al. (2025) [45], who conducted a systematic review and meta-analysis of 25 randomized controlled trials including 2577 participants. Exercise interventions, ranging from aerobic to resistance training, significantly reduced depression (*p* < 0.0001) and anxiety (*p* = 0.0002) in breast cancer survivors. Multicomponent training, examined in 16 studies, was identified as the most effective approach, particularly when performed at least three times per week in sessions lasting ≤60 min. In contrast to Zhang et al. (2025) [45], the present review establishes that multicomponent exercise programs should include at least three modalities out of aerobic, resistance, and either flexibility, balance, or pelvic floor training. This distinction provides a clearer framework for clinical translation and underscores the importance of structured and progressive programs that mirror real-world rehabilitation needs. Furthermore, by synthesizing dosage parameters (frequency, intensity, duration, and progression), the current review offers practical guidance that is often lacking in previous meta-analyses. This broader perspective, encompassing both active cancer patients and survivors, highlights the feasibility and additional benefits of comprehensive multicomponent interventions compared with protocols that incorporate only two exercise modalities.

Other approaches have also been evaluated. Correia et al. (2023) [46], in a meta-analysis of 28 randomized controlled trials with 2424 participants, reported that home-based exercise interventions during active cancer treatment significantly improved cardiorespiratory capacity as measured by the six-minute walk test. However, no significant changes were observed in strength or body composition, underscoring the importance of supervised interventions in this population.

Bowers et al. (2025) [47] reviewed 62 interventions in adults with cancer-associated cachexia and found that 52 percent of the programs included exercise components. These multicomponent interventions focused primarily on preserving physical function, with resistance training being the most used component.

In cancer survivors, Murnaghan et al. (2024) [48] identified exercise as a central component in six studies that implemented supervised aerobic, resistance, or group-based training programs. These interventions were associated with improvements in functional and psychosocial reintegration.

Regarding the feasibility of multicomponent exercise interventions for patients with stage I to III breast cancer undergoing active treatment, one study comparing radiotherapy and chemotherapy groups reported improvements in quality of life, body composition, and physical function, supporting the safety of these interventions [49].

### 4.1. Clinical Practice Implications

This review supports multicomponent exercise as a safe and effective adjunct therapy for patients with cancer, including those with advanced disease. To optimize outcomes, interventions should be individualized according to cancer type, disease stage, and clinical status. Programs must be structured and supervised, with exercise type and dose clearly defined during design. Integration of exercise into interdisciplinary cancer care has the potential to improve quality of life and functional performance in this population.

### 4.2. Research Implications

The findings underscore the need for studies with larger, well-defined populations and robust methodological designs to support the generalizability of multicomponent exercise effects in oncology, particularly in advanced or frail populations. Future research should include long-term follow-up to evaluate not only functional outcomes, pain, and quality of life but also disease progression, survival, and sustained adherence to exercise programs. It is also essential to incorporate pharmacological monitoring into study designs, given the potential impact of interactions between medical treatments and exercise interventions. Future studies should incorporate patient-centered outcomes. When assessing pain-related variables, a biopsychosocial framework is required, making constructs such as catastrophizing and kinesiophobia particularly relevant. Additional outcomes, including social participation, perceived well-being, feasibility, cost-effectiveness, and the personalization of exercise programs by cancer type and stage, should also be examined.

### 4.3. Limitations

This review has several limitations that should be considered when interpreting its findings. First, no firm conclusions can be drawn regarding the effects of multicomponent exercise on survival or pharmacological management. Most included studies did not report medication dosages, highlighting the need to account for concomitant treatments and specify which were used, particularly in patients with active cancer, to enable meaningful comparisons and assess potential treatment–exercise interactions. In addition, most studies lacked long-term follow-up and focused primarily on short-term outcomes such as physical function and quality of life. Although long-term follow-up is challenging given the prognosis of this population, it remains essential for future research.

Second, there was marked heterogeneity in the tools used to assess physical function (e.g., six-minute walk test, Short Physical Performance Battery, dynamometry) and pain. Standardization of pain assessment, for example, using the VAS for intensity and the BPI for impact, would improve comparability across studies. Finally, substantial variability was observed in intervention protocols. Future studies should adopt reporting checklists such as TIDieR [50] to ensure detailed description of exercise interventions. The limited number of trials directly comparing different modalities (e.g., multicomponent vs. aerobic-only exercise) also restricts conclusions about relative effectiveness. These limitations emphasize the need for studies with more homogeneous designs, longer follow-up, and more rigorous reporting. It is possible that, due to the search and information systematization process, some relevant studies were not accessible and therefore are not reflected in this review.

## 5. Conclusions

Multicomponent exercise shows beneficial effects on quality of life, depression, and physical function in patients with different cancer types, with mixed but promising evidence regarding pain outcomes. The most consistent benefits were observed in mobility, strength, and balance, including in advanced or metastatic disease and in vulnerable populations such as older adults and individuals with bone metastases. Programs combining at least three modalities—typically aerobic, resistance, and flexibility—implemented two to three times per week in 60–90-min sessions with progressive intensity adjustments appear particularly effective compared with passive or educational interventions. Although heterogeneity in study design remains a limitation and long-term effects on survival require further research, current evidence indicates that well-structured multicomponent interventions are safe, feasible, and clinically relevant even in complex oncological populations.

## Figures and Tables

**Table 1:** Characteristics of studies evaluating multicomponent exercise in cancer patients.

Author	Population/Mean Age	Type of Cancer	Stage	Intervention	Control	Pharmacological Treatment	Scales Used
Cheville et al. (2013) [29]	IG: N = 33, mean 63; CG: N = 33, mean 65	Colorectal, lung	IV	Program: aerobic, resistance with bands, flexibility	No prescribed exercise; booklet after study	NM	NRS, FACT-G
Reis et al. (2018) [30]	IG: N = 14, mean 47; CG: N = 14, mean 45	Breast	NM	12-week aerobic, resistance, flexibility training	Standard hospital care	NM	BPI
Mardani et al. (2021) [31]	IG: N = 35, mean 69; CG: N = 36, mean 70	Prostate	NM	Aerobic, resistance, flexibility, pelvic floor muscle exercises	Routine medical care; maintain usual physical activity/diet	NM	EORTC QLQ-C30
Wang et al. (2021) [32]	G: N = 23, mean 55; CG: N = 26, mean 56	Breast	NM	Personalized program: flexibility, wall push-ups, upper body resistance, warm-up/cooldown	Waitlist control with initial evaluation	NM	QoL instrument
Zimmer et al. (2018) [33]	IG: N = 17, mean 68; CG: N = 13, mean 70	Colorectal	IV	8-week supervised, endurance, Strength training, balance, coordination (2 week, 60 min)	Standard written recommendations	Chemotherapy (FOLFIRI, FOLFOX, oxaliplatin), Capecitabine, Bevacizumab, Regorafenib, Trastuzumab	FACT; GOG; h1RM; GGT-Reha; 6MWT
Levin GT et al. (2018) [34]	IG: EX: N = 10; SMHB: N = 8; CG: N = 14	Breast, Brain, Prostate, Kidney, Bladder	NM	EX: supervised resistance/aerobic, flexibility training 2/week; SMHB: self -managed ≥ 150 min/week aerobics. SMHB: received an exercise information booklet. Complete at least 150 min of aerobic exercise per week in sessions of 10 min or more	Only completing the questionnaire component of the study.	Chemotherapy and radiotherapy	HADS-D; HADS-A; GLTEQ; SF-36 (PHC and MHC); 400-m test
Liu et al. (2020) [35]	IG: N = 37; CG: N = 36	Lung: non-small cell lung carcinoma (NSCLC)	I–III: 89% in stages I–II, 11% in stage III	Aerobic exercise comprised the core activity of the intervention program. A 30-min home-based exercise of aerobic endurance exercise (jogging, walking, cycling, at discretion) was required for at least 3 days per week. Resistance exercise involving the major muscle groups (upper and lower limbs, chest and core muscles) was performed twice a week.	Usual clinical care	NM	WHODAS 2.0; 6MWD
Zopf et al. (2015) [36]	IG: N = 56; CG: N = 29	Prostate	II	15-month program: weekly 1-h sessions of aerobic, resistance, flexibility, coordination and pelvic floor exercises	No intervention	NM	EORTC-QLQ-C30; PCa (EORTC QLQ-PR25), Freiburger Questionnaire of Physical Activity.
Do et al. (2015) [37]	IG: N = 32; CG: N = 30	Breast	NM	Early/late groups: 4 weeks, 5/week, 80 min/day exercise (aerobic, strength, flexibility and core stability exercise)	Delayed exercise group receiving the same program starting after 4 weeks	NM	EORTC QLQ-C30; EORTC QLQ-BR23; FSS
Shinde et al. (2024) [38,39]	IG: N = 64, mean 50.9 ± 8.4; CG: N = 60, mean 49.7 ± 8.0	Breast	I–III	Phases 1–3 (12 months): progressive aerobic, strength (manual, isometric, resistance), flexibility, mobility, energy conservation strategies, and recreational therapy; home-based and supervised	Supportive therapy: health education, nutrition counseling, psychological counseling	Chemotherapy, radiotherapy, hormonal therapy (unspecified)	FACT-B
Ferrara et al. (2025) [40]	IG: N = 28, mean 74.4 ± 5.3; CG: N = 30, mean 74.4 ± 5.2	Colorectal, Upper digestive, Lung, Breast, Genitourinary, Head/Neck	III–IV	Supervised progressive resistance, balance training, walking (4 consecutive days, 2 sessions/day, 20 min each)	Usual care	NM	SPPB; Yesavage Geriatric Depression Scale; EORTC QLQ-C30; 1RM; Handgrip dynamometry
Fernández-Rodríguez et al. (2023) [41]	IG: N = 24; CG: N = 24, mean 63.5 ± 12.5	Lung, Digestive, Prostate, Breast, Others	II–IV	Aerobic, resistance, balance, moderate-load strength, flexibility; 2 sessions/day (15–20 min/session)	Standard care + educational lifestyle program	NM	VAS; Barthel Index; FACT; EuroQoL; SPPB
Haines et al. (2010) [42]	IG: N = 46, mean 55.9; CG: N = 43, mean 54.2	Breast	NM	Strength, balance, shoulder mobility, aerobic training	Flexibility and relaxation program	NM	EQ-5D; EORTC QLQ-C30; BR23
Galvão et al. (2018) [43]	IG: N = 28; CG: N = 29, mean 70	Prostate	IV	Supervised resistance, aerobic, flexibility (3x/week, 3 months)	Usual medical treatment	ADT: 95%; Chemotherapy: 16%	FACT-BP; SF-36; Self-reported physical function; 6MW; TUG
Zhang et al. (2023) [44]	IG: N = 92, mean 48.6; CG: N = 42, mean 49.3	Breast	NM	5-week hospital + home aerobic, strength, flexibility training	Usual care	NM	HADS; EORTC QLQ-C30

Abbreviations: IG = Intervention Group; CG = Control Group; NM = Not Mentioned; FACT-G = Functional Assessment of Cancer Therapy-General; BPI = Brief Pain Inventory; EORTC QLQ-C30 = European Organisation for Research and Treatment of Cancer Quality of Life Questionnaire; FACT = Functional Assessment of Cancer Therapy; GOG = Gynecologic Oncology Group; h1RM = one-repetition maximum for the bench press; GGT-Reha = German Guidelines for Rehabilitation in Oncology; instrument = Quality of Life instrument; EX = supervised exercise group; SMHB = Self-Managed Home-Based Exercise; HADS-D = Hospital Anxiety and Depression Scale- Depression; HADS-A = Hospital Anxiety and Depression Scale- Anxiety; GLTEQ = Godin Leisure-Time Exercise Questionnaire; SF-36 = Short Form Health Survey (36 items); PHC = Physical Health Component; MHC = mental Health Component; NSCLC = no small cell lung carcinoma; WHODAS 2.0 = World Health Organization Disability Assessment Schedule 2.0; 6MWD = Six-Minute Walk Distance; PCa (EORTC QLQ-PR25) = Prostate Cancer Module of the European Organization for Research and Treatment of Cancer Quality of Life Questionnaire-PR25; ADT = Androgen Deprivation Therapy; EORTC QLQ-BR23 = European Organization for Research and Treatment of Cancer Quality of Life Questionnaire-Breast Cancer Module (BR23); FACT-B = Functional Assessment of Cancer Therapy-Breast; SPPB = Short Physical Performance Battery; 1RM = One Repetition Maximum; VAS = Visual Analogue Scale; EQ-5D = EuroQol 5 Dimensions; FACT-BP = Functional Assessment of Cancer Therapy-Bone Pain; 6MW = 6-Minute Walk test; TUG = Timed Up and Go test; FSS: Fatigue Severity Scale.

**Table 2:** Pain, disability, quality of life and physical function outcomes in intervention and control groups.

Author	Pain (Instrument & Outcome)	Disability (Instrument & Result)	QoL (Instrument & Result)	Physical Function (PF) (Instrument & Result)	Key Notes
Cheville et al. (2013) [29]	NRS: IG −0.62 ± 2.59 CG −0.50 ± 2.01 (*p* = 0.87)	NM	Mobility: IG 4.88 ± 4.66 CG 0.23 ± 5.22 (*p* = 0.002); Activity: IG 1.56 ± 5.53, CG 0.94 ± 5.91 (*p* = 0.74) FACT-G: IG 1.07 ± 11.60 CG 0.12 ± 10.22 (*p* = 0.54)	NM	Pain unchanged; active mobility (QOL) improved in IG.
Reis et al. (2018) [30]	BPI: IG 2.43 ± 3.76 CG 3.93 ± 4.41 Total pain ↓ (*p* = 0.0047); general intensity (*p* = 0.0082); greater (*p* = 0.0284); lesser (*p* = 0.0365); interference in daily life (*p* = 0.0201)	NM	NM	VO_2_ max: IG 20.68 ± 2.50 CG 14.80 ± 2.46 (*p* = 0.0001) Handgrip right: IG 24.79 ± 6.77 CG 21.71 ± 7.44 (*p* = 0.0001)	↓ Pain; ↑ VO_2_max and handgrip strength in IG.
Mardani et al. (2021) [31]	EORTC QLQ-C30 Pain: IG 40.47 ± 16.31 *p* = 0.002, CG 28.24 ± 15.84	NM	EORTC QLQ-C30: IG Pre 60.19 ± 13.95 Post 72.57 ± 11.63 CG Pre 61.12 ± 14.10 CG Post 63.40 ± 12.80 *p* < 0.001		↓ Pain; ↑ Global QOL in IG.
Wang et al. (2021) [32]	NM	NM	FACT-ES IG: 151 ± 14 change +1 [−2, 4] CG: 142 ± 26 change −1 [−5, 3] *p* = 0.023	Steps/2 min IG 101 ± 23 change +18 [8, 28] CG 106 ± 23 change 9 [5, 13] *p* = 0.036	↑ QOL; ↑ Physical function in IG.
Zimmer et al. (2018) [33]	NM	NM	FACT-G: IG Pre 75.05 ± 14.82 IG Post 77.35 ± 11.83. CG Pre 76.42 ± 15.21 CG post 75.10 ± 14.95 *p* = 0.028	6 MWT distance: IG Pre 477.735 ± 91.911 IG Post 519.059 ± 68.958 CG Pre 459.654 ± 74.061 CG Post 482.154 ± 82.641 *p* = 0.432 h1RM [kg] leg press IG Pre 142.156 ± 44.125 Post 179.664 ± 68.196 *p* = 0.011 CG Pre 166.717 ± 56.304 159.991 ± 62.154	↑ QOL; ↑ Physical function in IG.
Levin GT et al. (2018) [34]	NM	NM	SF36 MHC Baseline: IG: 40.86 (9.82), SMHB: 43.84 (13.13), CG:44.09 (6.46) Week 6: IG: 44.23 (10.34), SMHB: 53.16 (5.75), CG: 41.94 (11.53) Week 12: IG: 51.63 (8.07), SMHB: 50.78 (9.87), CG: 40.85 (10.04) *p*: 0.005	400-m walk: Baseline: IG: 236.65 (40.43), SMHB: 251.39 (20.63) Week 6: IG: 226.48 (35.65), SMHB: 239.55 (22.77) Week 12: EX: 218.57 (32.75), SMHB: 231.01 (22.88) *p* = 0.466	↑ QOL (MHC) in IG.
Liu et al. (2020) [35]	NM	WHODAS 2.0a: Mean Difference Between Prehabilitation and Control Groups (95% CI): −1.0 (−2.4 to 0.4) *p*: 0.152	NM	6MWD (m): Mean Difference Between Prehabilitation and Control Groups (95% CI): 60.9 (32.4 to 89.5) *p*: < 0.001	↑ 6MWD in IG
Zopf et al. (2015) [36]	EORTC QLQ-C30 Pain IG: −2.37 points *p* = 0.679 CG: +3.33 points: *p* = 0.704	NM	EORTC QLQ-30: Change from Baseline to posttest IG: Mean 6.83 *p*: 0.064 CG: Mean 1.75 *p*: 0.717	EORTC QLQ-30: Change from Baseline to posttest IG: Mean 9.67 *p*: < 0.001 CG: Mean 4.20 *p*: 0.303	↑ Physical function in IG.
Do et al. (2015) [37]	EORTC QLQ-C30 Pain: IG pre 40.9 ± 28.1, IG post 19.4 ± 13.6, *p* < 0.001; CG no significant change	NM	EORTC QLQ-C30 IG pre 58.0 ± 18.6 IG post 87.3 ± 13.7 CG pre 59.5 ± 17.9 CG Post 61.0 ± 17.5 *p* = < 0.001	EORTC QLQ-C30 Physical function: IG pre 74.1 ± 17.3, IG post 89.4 ± 8.4, *p* < 0.001; CG similar later improvement	↑ QOL in IG.
Shinde et al. (2024) [38]	NM	NM	FACT-B: IG Pre 99.12 ± 10.24 IG Post 111.07 ± 11.61 CG Pre 100.05 ± 11.02 CG Post 102.15 ± 10.85 *p* = 0.001	NM	↑ QOL in IG.
Shinde et al. (2024) [39]	NM	NM	NM	STS: IG Pre 12.96 ± 4.24 IG Post 17.12 ± 7.39 *p* = 0.0002 CG Pre 13.86 ± 4.78 CG Post 14.2 ± 4.59 *p* = 0.92 12MWT IG Pre 1242.73 ± 205.68 IG post 1309.37 ± 167.35 *p* = 0.008 CG Pre 1249.91 ± 213.18 CG Post 1254 ± 186.59 *p* = 0.93	↑ PF in IG
Ferrara et al. (2025) [40]	NM	NM	EORTC QLQ-C30: IG 6.59, CG 4.93; Diff 1.65 (*p* = NS)	Leg press 1RM IG +9.54 (2.14–16.8) *p* = 0.153 Knee extension 1RM IG +9.77 (5.33 51.5) *p* = 0.018 SPPB IG 2.30 (1.43, 3.18) CG 0.38 (−0.34, 1.09) *p* = 0.002	No changes QOL; ↑ Knee extensor strength & balance in IG.
Fernández-Rodríguez et al. (2023) [41]	VAS: IG Pre 4.29 ± 2.21 IG Post 3.58 ± 2.78; EQ-5D Pain IG Pre: 1.96 ± 0.69 IG Post 1.71 ± 0.62 (*p* = 0.334)	NM	FACT: IG Pre 94.96 ± 11.91 IG Post 102.75 ± 13.00 CG Pre 95.10 ± 11.70 CG Post 96.00 ± 11.50 *p* = 0.036	SPPB total IG 6.21 ± 2.99 CG 4.42 ± 3.03 *p* = 0.045 SPPB gait IG 1.75 ± 0.94 CG 1.17 ± 0.91 *p* = 0.035	↑ QOL (follow-up IG); ↑ PF.
Haines et al. (2010) [42]	EQ-5D VAS: IG 80.6, CG 74.1 comparison 10.08 (2.84, 17.32) (*p* = 0.006)	NM	EORTC QLQ-C30 IG Pre 69.4 ± 15.1 IG Post 76.6 ± 15.0 CG Pre 70.0 ± 14.9 CG Post 71.2 ± 15.1 *p* = 0.04	6MWT (m) IG 535 ±110, CG 532 ± 82 comparison 22.52 (−23.24, 68.28), *p* = 0.34 Grip strength (kg) IG 25.4 ± 7.7 CG 24.2 ± 5.4, comparison −1.24 (−4.65, 2.17) *p* = 0.48	↑ QOL in IG; no change in PF.
Galvão et al. (2018) [43]	FACT-BP: *p* = 0.507	NM	NM	Leg extension 1RM IG +6.6 kg (95% CI 0.6 12.7) *p* = 0.033	No change in pain; ↑ PF.
Zhang et al. (2023) [44]	NM	NM	EORTC QLQ-C30 FF, FE, SG, Fatigue, Insomnia (all *p* < 0.05)		↑ QOL in IG

Abbreviations: IG: Intervention Group; CG: Control Group; NM: Not Measured; NRS: Numeric Rating Scale; BPI: Brief Pain Inventory; VO_2_ max: Maximal Oxygen Uptake; EORTC QLQ-C30: European Organisation for Research and Treatment of Cancer Quality of Life Questionnaire–Core 30; FACT-G: Functional Assessment of Cancer Therapy–General; FACT-ES: Functional Assessment of Cancer Therapy–Endocrine Symptoms; 6MWT: 6-Minute Walk Test; 1RM: One Repetition Maximum; SF-36: Short Form Health Survey (36 items); WHODAS 2.0: World Health Organization Disability Assessment Schedule 2.0; SPPB: Short Physical Performance Battery; STS: Sit-to-Stand Test; 12MWT: 12-Minute Walk Test; FACT-B: Functional Assessment of Cancer Therapy–Breast; VAS: Visual Analogue Scale; EQ-5D: EuroQol 5 Dimensions; FACT-BP: Functional Assessment of Cancer Therapy–Bone Pain; MHC: Mental Health Component; SMHB: Supervised Mind–Body group; FF: Functional functioning; FE: Financial functioning; SG: Social functioning.

## Data Availability

No new data were created or analyzed in this study. Data sharing is not applicable to this article.

## Appendix A. Search Terms

**Search Combination Terms**
Exercise therapy AND Cancer
Exercise multicomponent AND Cancer pain
Exercise multicomponent AND neoplasm
Exercise therapy AND Cancer pain
physical training AND Cancer Pain
Exercise therapy AND neoplasm
Exercise multicomponent AND Chemotherapy
Exercise multicomponent AND Radiation therapy
Exercise multicomponent AND Palliative therapy
